# Screening for Mild Cognitive Impairment: There is the Will but Is There a Way?

**DOI:** 10.14283/jpad.2020.16

**Published:** 2020

**Authors:** J.E. Galvin

**Affiliations:** Comprehensive Center for Brain Health, Department of Neurology, University of Miami Miller School of Medicine

Alzheimer’s disease (AD) currently affect over 5.8 million Americans and over 35 million people worldwide (1). The number of AD cases is expected to increase as the number of people over age 65 grow by 62% and the number over age 85 is expected to grow by 84% (1, 2). More than one in eight adults over age 65 has dementia, and current projections indicate a three-fold increase by 2050. Thus, the prevalence, incidence, morbidity, and mortality for AD (3) and its prodromal state of Mild Cognitive Impairment due to AD (MCI-AD) (4) will increase dramatically and the societal financial burden of illness and dependency will expand exponentially. Primary care providers are often responsible for the detection, diagnosis, and treatment of AD as the number of dementia specialists (neurologists, psychiatrists, and geriatricians) and specialty centers is not sufficient to meet the growing demands (2, 5). The inability to detect MCI and ADRD may affect eligibility determination for care and services and impede case ascertainment and recruitment in clinical research.

Despite these obvious demands for early detection, MCI-AD is often under-recognized in community practice, with many individuals obtaining a diagnosis in the mild-to-moderate stages of dementia, particularly in older adults from underrepresented and underserved communities (2, 5). MCI-AD screening would increase case identification; however, there are questions as to whether increased screening and case identification have value in the absence of more effective interventions that can improve patient outcomes (6).

In recognition of current challenges around the detection of MCI-AD, an international expert working group was convened in April 2019 to describe the opportunities and challenges to make a diagnosis of MCI-AD in the clinic and home settings. This conference led to a series of three papers in the current issue of Journal of Prevention of Alzheimer’s Disease (7–9). The first paper discusses pros and cons of early detection based on current research and considers how the changing landscape of technology and scientific advances will alter these perceptions (7). The second paper discussing methods for MCI-AD screening in the primary care setting, where most patients are encountered (8). The third paper offers thoughts on how MCI-AD screening could potentially be done in the home setting, with reports being made available to primary care providers at office visits (9).

MCI-AD screening is ideally suited for the primary care setting as the providers typically have long standing relationships with patients, have more readily available appointment slots, are already following other chronic conditions, and can provide continuity of care (2, 10). In particular, the Medicare Annual Wellness visit was designed to take advantage of this setting and has assessment of cognitive function as a requirement. However only 20% of Medicare beneficiaries have an Annual Wellness Visit and there are no clear guidelines as to what constitutes a cognitive assessment (1). In primary care settings, this is unlikely to include the detailed neuropsychological tests or invasive biomarkers often used in research settings or at University-based memory disorder centers.

A Special Report by the Alzheimer’s Association “Alzheimer’s Detection in the Primary Care Setting” (1) focused on early AD and MCI detection in the primary care setting (which constitutes 1/3 of medical workforce in the US), where the majority of patients are seen and the first point of contact for most individuals. Particularly informative were the results of two surveys: (a) 1000 primary care providers and (b) 1954 older adults regarding expectations, benefits and practices about AD and MCI screening. While 94% of patients saw their primary care providers in the last year, only 47% discussed memory and 28% received a memory assessment. This contrasts with 95% of older adults wanting to know about their memory and 51% reporting changes. Providers reported that 50% assess cognition as part of their evaluation but only 40% are familiar with the toolkits available to them. For those that do assess cognition, only 64% informed the patients of the results. This contrasts with more than 90% of primary care providers reporting there are benefits to dementia screening including advanced care planning and interventions (1).

This series of papers offers a compelling argument for thinking “outside the box” to increase the likelihood of early detection of MCI-AD and provide potentially actionable methods such as creation of new tools and screening platforms, computerized testing, integration of screening tools into electronic health records, and offering in-home testing that does not require any physician or staff time. If the long-term goal is to increase “real world” early MCI and AD detection, diagnosis, and treatment, improve patient care, reduce disparities in health outcomes particularly in underserved communities, and reduce healthcare costs then we must take a transformative approach in our thinking about how best to serve our patients and the communities in which they live. This resonates strongly with the three guiding principles of the National Alzheimer’s Project Act (NAPA) (11), especially its third principle: “Transform the way we approach Alzheimer’s disease and related dementias.”
